# The Neglected Tropical Diseases of India and South Asia: Review of Their Prevalence, Distribution, and Control or Elimination

**DOI:** 10.1371/journal.pntd.0001222

**Published:** 2011-10-25

**Authors:** Derek A. Lobo, Raman Velayudhan, Priya Chatterjee, Harajeshwar Kohli, Peter J. Hotez

**Affiliations:** 1 Department of Public Health, Manipal University (Retired – WHO-South-East Asia Regional Office), Mangalore, India; 2 Department of Control of Neglected Tropical Diseases, World Health Organization, Geneva, Switzerland; 3 George Washington University, Washington, D.C., United States of America; 4 Sabin Vaccine Institute and Texas Children's Center for Vaccine Development, and the National School of Tropical Medicine at Baylor College of Medicine, Houston, Texas, United States of America

## Introduction

The neglected tropical diseases (NTDs) are the most common infections of the world's poorest people living in Africa, Asia, and the Americas [1]. Occurring predominantly among people who live on less than US$2 per day or below the World Bank poverty figure of US$1.25 per day, the NTDs represent a group of chronic parasitic and related bacterial and viral infections that actually promote poverty because of their impact on child development, pregnancy outcome, and worker productivity [2]. The NTDs differ significantly in their prevalence and disease burden according to their geographic and regional presence. Such features for the NTDs in sub-Saharan Africa [3], China and East Asia [4], and the Americas [5]–[7], respectively, were reviewed previously. Here, we summarize current knowledge on the prevalence, distribution, and disease burden of the NTDs in India and South Asia, focusing on aspects particular to the region. The review of the literature was conducted using the online database PubMed from 2003 to 2010 with the Medical Subject Headings, the specific diseases listed in the World Health Organization's (WHO) first report on NTDs [8], and the geographic regions and countries of South Asia. Reference lists of identified articles and reviews were also hand searched as were WHO databases (http://www.who.int/), including the WHO's *Weekly Epidemiological Record*. Recently, a comprehensive review on the continuing challenge of infectious diseases in India was published [9]. However, this review focuses exclusively on NTDs, many of which, especially the helminthiases, were not emphasized previously [9].

## Overview of NTDs in India and South Asia

**Figure 1:**
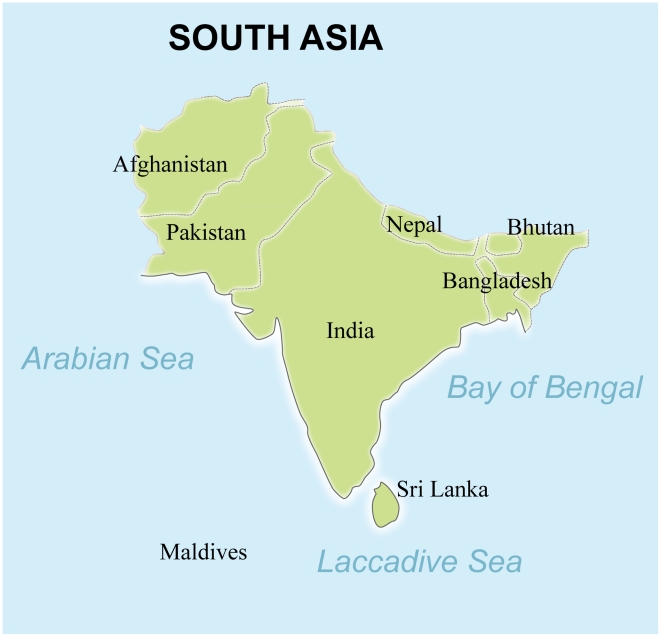
South Asia. Map created by Priya Chatterjee, The George Washington University, using Arc GIS version 9.3.1.

There is no single and universally accepted definition of the geographic area known as South Asia; however, most definitions include the nations of Bangladesh, India, Maldives, Nepal, and Sri Lanka. The WHO South-East Asian region also adds DPR Korea, Indonesia, Myanmar, Thailand, and Timor-Leste (http://www.searo.who.int/). Because the prevalence and disease burden of the major NTDs in East Asia were previously reviewed and included those five countries [4], we instead adopted the World Bank's use of the term *South Asia*, which incorporates the eight nations of Afghanistan, Bangladesh, Bhutan, India, Maldives, Nepal, Pakistan, and Sri Lanka [10], [11] (Figure 1). With a few exceptions, very little data on the NTDs are available from Afghanistan, so the information provided here emphasizes the NTDs in the other seven countries.

Together, the South Asian nations mentioned above represent a population of 1.5 billion, or almost one-quarter, of the global population [11]. The major countries and their populations are listed in Table 1, with India accounting for 75% of the number of people living in South Asia. Although the World Bank reports that South Asia has experienced an impressive economic rebound since the global recession in 2009, with approximately 7% overall economic growth in 2010 [11], this rising tide has left behind a substantial number of people who remain trapped in poverty. Today over 1 billion people in South Asia live on less than US$2 per day [10], [11]. Moreover, the prevalence of underweight children in South Asia exceeds 40% in India, Bangladesh, and Pakistan, where the rates of malnutrition are considered among the highest in the world and are nearly double that of sub-Saharan Africa [10], [11]. As shown in Table 2, the 1 billion or so South Asians living in poverty suffer from high rates of NTDs. Today, South Asia accounts for approximately one-quarter of the world's soil-transmitted helminth infections, one-third or more of the global deaths from rabies, and one-half or more of the global burden of lymphatic filariasis, visceral leishmaniasis, and leprosy. The region is also experiencing an emerging problem with three major arbovirus infections, i.e., dengue, Japanese encephalitis, and Chikungunya. For several other important NTDs, such as strongyloidiasis, toxocariasis, leptospirosis, and amebiasis, there are no prevalence or disease burden estimates available.

**Table 1:** The Countries and Population of South Asia and the Percentage Living in Poverty.

Nation	Total Population^a^	Percentage of Population Living Below US$1.25 per Day in 2009^b^
India	1.13 billion	41.6%
Pakistan	166 million	22.6%
Bangladesh	160 million	49.6%
Nepal	28 million	55.1%
Afghanistan	28 million	Not available
Sri Lanka	20 million	14.0%
Bhutan	0.7 million	26.2%
Maldives	0.3 million	Not available
Total South Asia	1.53 billion	

a From [10], [11].

b Asian Development Bank key indicators for Asia and the Pacific.

**Table 2:** The Major NTDs in India and South Asia Ranked by Prevalence.

Disease	Number of Cases in India (Percentage of Global Disease Burden)	Number of Cases in India and South Asia (Percentage of Global Disease Burden)	Estimated Number of DALYs in South Asia^c^	Reference
Ascariasis	140 million (17%)	237 million (29%)^a^	0.4–3.0 million	[12], [13]
Trichuriasis	73 million (12%)	147 million (24%)^a^	0.5–1.5 million	[12], [13]
Hookworm infection	71 million (12%)	130 million (23%)^a^	0.6–5.6 million	12,13
Lymphatic filariasis	<6 million (5%) (based on 0.53% prevalence)	<60 million (50%)^b^	2.9 million^b^	[22]
Trachoma	1 million (1%–2%)	2 million (2%–4%)^a^	<0.1 million	[37], [38]
Visceral leishmaniasis	Not determined	200,000–300,000 cases (40%–60%)	0.4–1.0 million	[26], [27]
Leprosy	87,190 registered cases (41%)	120,456 registered cases (57%)^b^	0.1 million	[35]
Rabies	20,000 cases/deaths (36%)	≥20,000 cases/deaths (>36%)	Not determined	[8], [48]
Japanese encephalitis	1,500–4,000 (incidence)	1,000–3,000 (incidence, Nepal); 100–200 (incidence, Sri Lanka)	0.3 million^b^	[47], [50]
Dengue	Not determined	Not determined	0.4 million^b^	[47], [50]
Total			5.6–14.8 million	

a World Bank South Asia Region: Afghanistan, Bangladesh, Bhutan, India, Maldives, Nepal, Pakistan, Sri Lanka.

b WHO South-East Asia Region: Bangladesh, India, Indonesia, Maldives, Myanmar, Nepal, Sri Lanka, Thailand, Timor-Leste.

c DALYs lost from NTDs in South Asia calculated on the basis of the DALYs estimated in references [2], [51] multiplied by the percentage of cases in South Asia, with the exception of the DALYs for dengue and Japanese encephalitis, which were quoted directly from [50].

## Helminth Infections

The major helminth infections in South Asia include three soil-transmitted helminth infections, i.e., ascariasis, trichuriasis, and hookworm infection (“hookworm”), and lymphatic filariasis.

### Soil-Transmitted Helminth Infections

These helminthiases represent the three most common NTDs in South Asia. Ascariasis (*Ascaris lumbricoides* infection) is the most common helminth infection and NTD in the region, with more than 200 million cases, followed by more than 100 million cases of trichuriasis (*Trichuris trichiura*) and hookworm, respectively [12], [13]. Whereas *Necator americanus* accounts for most of the world's cases of human hookworm infections, in Uttar Pradesh and West Bengal States, and presumably elsewhere in India, mixed infections with both *N. americanus* and *Ancylostoma duodenale* also occur, as well as pure *A. duodenale* infections [14]. *A. duodenale* has also been identified as a cause of infantile hookworm [15]. In Pakistan, wastewater used in agriculture was found to be a significant risk factor for hookworm [16]. Overall, South Asia accounts for approximately one-quarter of the world's cases soil-transmitted helminthiases, with the largest number of cases in India, followed by Bangladesh. These numbers are based on data published in 2003 [13]; more recent data from the Global Atlas of Helminth Infections [17] are not yet available for South Asia.

Because of their pronounced impact on child growth and development, in 2001 the 54th World Health Assembly established a target to reduce the prevalence and the intensity of soil-transmitted helminth infections in all countries by 50% and achieve a target of regular deworming of at least 75% of school-age children at risk [8]. The major strategy relies on once or twice yearly mass drug administration (MDA) using the drug mebendazole or albendazole as a single dose, with a drug delivery system relying heavily on schools and schoolteachers administering the drugs. Among school-aged children only Bhutan has achieved this target to date, although approximately one-half of Sri Lanka now receives regular deworming in national control campaigns [18]. However, a higher percentage of pre-school-aged children receive deworming, especially in Bangladesh, India, Nepal, and Sri Lanka, possibly because children receive single-dose albendazole as part of lymphatic filariasis (LF) elimination efforts that combine MDA with this drug together with diethylcarbamazine citrate (DEC). In addition, Nepal has been targeted for helminth control, together with LF and trachoma elimination efforts, through a United States–supported NTD Program [19], while in Sri Lanka the overall prevalence of soil-transmitted helminth infections among school-aged children falls below the WHO-recommended level required for annual deworming [20]. A human hookworm vaccine is also under development to prevent post-treatment re-infection [21].

### Lymphatic Filariasis (LF)

LF is one of the most debilitating and disfiguring diseases in South Asia, where almost all of the cases are caused by *Wuchereria bancrofti* [9], [22]. The adult worms inhabit the lymphatics, which in late stages lead to lymphoedema and elephantiasis. The disease is poverty-related and predominantly affects poor and marginalized groups [23]. LF-associated disabilities and deformities result in heavy economic losses and loss of livelihood [24].

The WHO South-East Asian region (which also includes the LF-endemic countries of Indonesia, Myanmar, Thailand, and Timor-Leste) accounts for the single highest disease burden of LF, with approximately 50% of the estimated 120 million cases globally and 67% of disease burden when measured in disability-adjusted life years (DALYs) [22]. India alone has 40% of the LF global disease burden [9]. There is also a huge socioeconomic impact [2] due to impaired worker productivity resulting from lymphoedema of the lower limbs and hydrocele [23], [24]. India loses almost US$1 billion annually from LF [24], while in a recent qualitative study in Sri Lanka, Perera et al. [23] have also articulated LF's social stigma. In South Asia, the nations of Bangladesh, India, Maldives, Nepal, and Sri Lanka are endemic for LF [22].

LF is targeted by WHO for elimination as a public health problem, defined as a microfilaraemia rate of <1%. In 1997, the World Health Assembly passed a resolution to work towards LF elimination, and in 2000 the WHO's Global Programme to Eliminate LF established a goal to eliminate the infection by 2020 [8], [22]. The main strategies are: 1) annual MDA with two drugs, DEC and albendazole, to the entire eligible population for 5–6 years, and 2) home-based disability alleviation and prevention [8], [22]. To date, Sri Lanka has completed and stopped MDA, while India has implemented MDA with almost 100% geographical coverage of its endemic areas [22]. India's National Vector Borne Disease Programme for LF elimination is impressive by its sheer scale and scope [25]. Today, with treatments offered to the entire endemic population of 600 million people, MDA for LF in India is that country's largest national public health intervention [22]. The overall prevalence of microfilaremia for LF was cut in half between 2004 and 2008 and today the prevalence is 0.53% [22]. Bangladesh, Maldives, and Nepal are also implementing MDA with high rates of coverage [22].

## Protozoan Infections

Leishmaniasis and amebiasis represent the highest burden protozoan NTDs.

### Visceral Leishmaniasis (VL)

Also known as kala-azar, an estimated 200,000–300,000 people are infected in South Asia, representing more than 60% of the world's cases of VL [26], [27]. Many of South Asia's VL cases occur in contiguous areas of Bangladesh, India, and Nepal [27]; in India VL is found primarily in the state of Bihar, as well as in some neighboring districts in Uttar Pradesh, and in West Bengal [28]. In South Asia, VL is caused by *Leishmania donovani* and transmitted to humans by the bite of an infected female sandfly, *Phlebotomus argentipes.* VL lowers immunity, causes persistent fever, pancytopenia, and enlargement of the spleen and liver, and leads to very high mortality in untreated cases. Post kala-azar dermal leishmaniasis (PKDL) is also an important complication. In this condition, numerous parasites are lodged in the lesions in the skin, creating a chronic source for further transmission. VL is also an important opportunistic infection of patients with HIV/AIDS [29].

In South Asia VL is mainly a rural disease predominantly affecting the poor, and poverty is a key determinant of this disease [26], [30]. Among the risk factors that promote survival of the insect vector and foster disease transmission are mud walls, dampness in houses, and peridomestic vegetation [26]. It has also been noted that women often delay seeking VL treatments and are more likely to die from their infection [30]. Even though VL in South Asia is anthroponotic (there is no significant animal reservoir), in some studies the presence of cattle is associated with an increased risk of acquiring the infection [26], [30]. VL cases tend to cluster at the household level and entire villages can become infected during a VL epidemic over a short period (which is then often followed by an outbreak of PKDL cases) [26]. Like many NTDs, VL may actually promote poverty because of its impact on children and worker productivity [30]. In addition, the families of VL patients must often use a significant percentage of their earnings or savings for often expensive treatments. The high cost is a particular problem in the impoverished state of Bihar where antimonial drug resistance is high and the alternative treatments, especially liposomal amphotericin B, are often prohibitively expensive [26], [30].

VL is being targeted by the WHO for elimination in South Asia [26], [27], defined as an incidence of <1 case per 10,000 population at each endemic district. The elimination goal received a boost in 2000 when the ministers of health of Bangladesh, India, and Nepal met in Kathmandu, Nepal, under the auspices of the WHO, endorsed a joint action strategy for this goal, which includes an administrative commitment to eliminate VL by 2015 [27]. This joint action is essential, based in part on the finding that 50% of VL cases occur in the border districts of these three countries [27]. Following the ministerial meeting, a draft regional strategic plan was developed and endorsed by the three countries during an inter-country meeting held in Varanasi, India, in November 2003. The plan was reviewed by the Regional Technical Advisory Group (RTAG) for kala azar held in India, December 2004, and was finally adopted by the national governments and partners at a meeting in India, in August 2005. The major elements of the strategy include: 1) early diagnosis wherever possible, with the rapid diagnostic test rk-39 and prompt treatment with the oral drug miltefosine, injectable paromomycin, or liposomal amphotericin B [26], [27]; 2) integrated vector management, which includes bed nets and indoor residual spraying with DDT and other agents [9], [26]; 3) effective disease surveillance; 4) social mobilization and partnerships; and 5) clinical and operational research [27], [31]. Among the challenges to VL elimination in South Asia are the high rates of PKDL—PKDL patients represent a potent source for *Leishmania* parasites and require a prolonged treatment period [26]. Several candidate vaccines to prevent VL are also under development [32].

In addition to the problem of VL, Afghanistan has experienced a re-emergence of disfiguring cutaneous leishmaniasis (CL), especially in Kabul [8]. Conflict and its association with a weakened health care infrastructure combined with environmental degradation are key factors believed to be responsible for this resurgence [33].

### Amebiasis

Amebiasis is another important protozoan infection, especially in India and Bangladesh, although there are minimal surveillance data available and no known disease burden information. Among the difficulties in elucidating the extent of this infection is the absence of widespread testing to differentiate amebiasis caused by pathogenic *Entamoeba histolytica* versus the non-pathogenic *Entamoeba dispar* [34].

## Neglected Bacterial Infections

The major neglected bacterial infections in South Asia include leprosy, trachoma, and leptospirosis.

### Leprosy

Caused by *Mycobacterium leprae*, leprosy is one of the oldest diseases known to humankind. The disease primarily affects skin and peripheral nerves, which can lead to crippling deformities of the hands, feet, and face if left undiagnosed or untreated. The disease disproportionately affects the poor and other vulnerable and marginalized population groups; its victims are often exposed to stigma, prejudice, discrimination, and ostracism. With the implementation of multi-drug therapy (MDT), a combination of three drugs promoted by WHO since the early 1980s, there has been a dramatic decline in global leprosy cases—from >12 million cases in 1985 to <0.25 million in 2009 [35]. Encouraged by the success of MDT, in 1991, the World Health Assembly passed a resolution to work towards the elimination of leprosy as a public health problem, defined as a prevalence of <1 case per 10,000 population [8]. In 1985, there were 122 leprosy-endemic countries with a national prevalence of >1/10,000 population. By the end of 2010, 121 of the 122 countries (Brazil being the only exception) have achieved the leprosy elimination goal at the national level and several of them have also achieved the goal at the sub-national level. The Global Leprosy Programme is thus one of the outstanding success stories in public health.

Some of the greatest gains in terms of leprosy elimination have occurred in the WHO's South-East Asian region. Among the key factors that contributed to this success are: 1) strong political commitment and allocation of resources by national governments; 2) a free supply of anti-leprosy drugs from WHO, thanks to the generous grants from the Nippon Foundation and the Novartis Trust for Sustainable Development; 3) the leadership provided by WHO and effective coordination with national programs and partners; and 4) strong partnerships involving the World Bank, other United Nations (UN) agencies, international/national nongovernmental organizations, and support of key groups like media, religious leaders, local community leaders, and youth/women's groups. Currently, of the world's 212,000 registered cases of leprosy, more than one-half still occur in South Asia [8]. Nepal was the last country in the region to achieve the leprosy elimination goal in 2010. India accounts for 40% of the world's registered cases and for more than one-half of the almost 250,000 new leprosy cases detected annually [35]. The following factors have been identified in ensuring success in leprosy elimination efforts in South Asia: 1) sustaining political commitment and ensuring adequate resources, with progress towards further reducing the burden of leprosy at sub-national levels, particularly in large countries like Bangladesh and India; 2) strengthening integration of leprosy services into the general health system through capacity building and skill development, in order to ensure and sustain quality leprosy services, including diagnosis and treatment at all levels—this factor has been cited as a key reason for gains in India's leprosy elimination efforts [36]; 3) ensuring a wider coverage of leprosy services, especially in currently under-served population groups such as remote rural areas, urban slums, and migrant labor; 4) increasing and sustaining community awareness through advocacy activities to promote voluntary case detection and decrease the stigma; 5) prevention of the care of disabilities and displacement of leprosy-affected individuals and ensuring community-based rehabilitation of cured/disabled leprosy persons; and 6) streamlining the MDT supply and stock management at all levels, especially in areas of low endemicity.

### Active Trachoma

Worldwide, trachoma is a leading cause of visual impairment and blindness. According to the WHO's world trachoma atlas using data from 2003, approximately 1 million cases of trachoma occur in India, particularly in Rajasthan [37], and 200,000–300,000 cases in Afghanistan, Nepal, and Pakistan [38]. These cases represent less than 5% of the world's trachoma disease burden [39]. However, other sources indicate that India may account for a much larger contribution to the global trachoma disease burden [8], [37].

### Leptospirosis

Although leptospirosis is believed to be an important NTD in South Asia, there is a paucity of prevalence and disease burden information. However, because of its association with flooding, leptospirosis is believed to be an important cause of acute febrile illness in children and aseptic meningitis, especially in the monsoon and immediate post-monsoon seasons [40]. The disease is endemic in the Indian states of Kerala (where the seroprevalence is especially high among high-risk groups such as sewage workers, hospital sanitary workers, and fisherman), Tamil Nadu, and the Adamans, and outbreaks are common in the slums of Mumbai [40].

## Neglected Viral Infections

The major neglected viral infections in India and South Asia include the two major arboviral infections, dengue and Japanese encephalitis, and rabies.

### Dengue

The first “virologically proven” epidemic of dengue in India occurred in Kolkata and the Eastern coast in 1963–1964, subsequently reaching the entire country with all four dengue serotypes [41]. However, at least a dozen other epidemics of a dengue-like illness were recorded throughout the 19th and 20th centuries [41]. Dengue hemorrhagic fever was first reported from India only in 1987, with a large outbreak occurring in Delhi in 1996 [9], [41]. Although initially a largely urban disease, dengue has now spread to rural areas [41]–[43], with dengue cases occurring throughout the year [41]. In Bhutan and Nepal, dengue was first reported in 2004 [44], [45]. Overall, in WHO's South-East Asia region the number of severe dengue cases has increased since 2006 [8]. Dengue continues to be reported in all countries of South Asia and sustained vector control efforts need to be initiated.

### Japanese Encephalitis (JE)

JE is believed to have been introduced to South Asia from East Asia within the last half of the 20th century [46]. As a result of its recent emergence in the region, JE affects both children and adults in northern India, Nepal, and Sri Lanka, whereas it is predominantly a pediatric disease in the Asia-Pacific region [46]. Large epidemics in northern India and Nepal occur primarily during the summer months [47]. Although JE can be a highly fatal disease, most individuals are asymptomatic. Due to the absence of vaccination programs and possibly other interventions, the incidence of JE in Bangladesh, India, and possibly Pakistan was noted previously to be on the rise, whereas it had decreased in Nepal and Sri Lanka, where both surveillance and vaccination programs are in place [47]. Today, Bangladesh, India, and Pakistan exhibit the highest JE disease burden in South Asia [47]. Two key factors responsible for JE emergence in South Asia include population growth and irrigated rice farming, which creates suitable breeding sites for mosquito vectors [47]. Climate change may also represent an important factor. In addition to the vaccination programs in Nepal and Sri Lanka, the Indian Ministry of Health has recently developed plans for surveillance and national vaccination of children; immunization programs have begun in both Tamil Nadu and Uttar Pradesh [47]. More than 9 million children were vaccinated in India in 2006, and since then vaccination programs have been introduced into all 62 endemic districts [9].

### Chikungunya

Chikungunya was first identified in Tanzania in the early 1950s and has caused periodic outbreaks in Asia and Africa since the 1960s. It is rarely fatal. Significant pain occurs in the joints and the pain can persist for several weeks. Chikungunya shares some clinical signs with dengue and can be misdiagnosed in areas where dengue is common. Between 2001 and 2007, a number of countries reported Chikungunya outbreaks. In an outbreak in India in 2006, 1.4 million cases were reported (although the number of actual cases is believed to be considerably higher) with *Aedes aegypti* implicated as the vector [9].

### Rabies

Rabies is an important neglected zoonotic disease in South Asia. Canine rabies is enzootic in India and it is estimated that India accounts for 36% of the world's deaths from rabies (approximately 20,000 or more), with between 30% and 60% occurring in children, and most of the cases in rural areas [48]. Almost all of these deaths are preventable through prompt medical attention comprised of wound cleaning and care and post-exposure prophylaxis with rabies vaccine. It is estimated that the canine population of India is as high as 25 million [48], which makes a national program of canine mass vaccination difficult even though it is considered one of the most cost-effective ways to reduce human rabies deaths [49]. In 2008, an Indian pilot project to prevent human rabies deaths was launched by the National Centre for Disease Control in five Indian cities. The project includes programs to increase awareness by the public and health care professionals about the importance of immediate medical attention to animal bites and scratches [48]. In addition, Sri Lanka has made great strides in eliminating dog rabies, while Nepal is producing its own rabies vaccines for humans and dogs [8]. Throughout the affected enzootic countries it was recommended that comprehensive national rabies control programs should be established [49].

## Concluding Remarks

Together, the NTDs result in an estimated 5.6–14.8 million DALYs lost annually (Table 2)—this number exceeds the DALYs lost annually in the WHO South-East Asian region as a result of malaria, while the higher value is approximately the same as the DALYs lost from HIV/AIDS or tuberculosis [50]. Comprehensive programs to eliminate some of the highest prevalence NTDs are under way in South Asia. They include activities of the Global Programme to Eliminate LF, which is conducting national programs of MDA, together with an international VL elimination effort emphasizing the large number of cases occurring in the border areas of Bangladesh, India, and Nepal, and national programs of MDT for leprosy. Although JE has recently emerged in South Asia, it may also be controlled or eliminated through national programs of comprehensive vaccination.

For other NTDs, national control programs of preventive chemotherapy, especially MDA for trachoma (in conjunction with SAFE strategies) and soil-transmitted helminth infections, and efforts to vaccinate against canine rabies (as well as cholera), need to be expanded. Such programs require integration with improvements in sanitation and access to clean water. Integrated vector management that combines bed nets with insecticides are key elements for the control of VL, CL, and the arbovirus infections. Among the new control tools under development that could facilitate NTD and other disease elimination efforts are new or improved vaccines under development for cholera, dengue, hookworm infection, leishmaniasis, and malaria [20]. There is an urgent need for better surveillance and disease burden assessments for most of the NTDs, but especially for amebiasis, leptospirosis, and the major arbovirus infections, and for linking MDA, vaccinations, integrated vector management, and improved surveillance together as part of overall efforts to strengthen health systems in the region.
